# Cutaneous Lesions in Freshwater Turtles (*Emydura macquarii krefftii* and *Myuchelys latisternum*) in a Rainforest Creek in North Queensland, Australia

**DOI:** 10.3389/fvets.2020.00033

**Published:** 2020-01-31

**Authors:** Wytamma Wirth, Elizabeth Elliott, Donna Rudd, Linda Hayes, Alicia Maclaine, Narges Mashkour, Shamim Ahasan, Jakob Gorm Dahl, Kezia Drane, Ellen Ariel

**Affiliations:** ^1^College of Public Health, Medical and Veterinary Sciences, James Cook University, Douglas, QLD, Australia; ^2^AusPhage, Rasmussen, QLD, Australia; ^3^Faculty of Veterinary and Animal Sciences, Hajee Mohammad Danesh Science and Technology University, Rangpur, Bangladesh

**Keywords:** freshwater turtles, cutaneous lesions, disease, wildlife, outbreak

## Abstract

Freshwater turtles inhabit most rivers and creeks on the east coast of Australia, but some species are only found in specific catchments, which makes them vulnerable to extinction. During annual fieldtrips to Alligator Creek, North Queensland, the resident population of *Myuchelys latisternum* and *Emydura macquarii krefftii* in a natural pond, just outside Bowling Green National Park, have been surveyed for a number of years and demographic data recorded against tagged turtles. Rounded, cutaneous lesions on individual animals were first noted in August 2016, three years after the first survey of the population. Turtles living in the upstream sections of the creek were not affected. An initial investigation into the cause of the lesions ruled out pollutants and although the bacterial communities appeared to be different on turtles with lesions, a causative agent was not identified. Attempts to isolate virus in culture was not successful and specific PCRs for ranavirus, papillomavirus, adenovirus and herpesvirus did not identify their presence. Blood biochemical parameters, body condition and activity levels were not significantly different between affected turtles and those without lesions. The turtles in this pond were monitored regularly over the following three years with 249 *M. latisternum* and 192 *E. m. krefftii* captured, tagged and released. The prevalence of the lesions fluctuated with season from 0 to 77 and 68% respectively, but did not vary significantly between species or sex in adults. There was a tendency for larger animals to be more likely to have lesions. The position of the lesions on the turtles was mostly on dorsal surfaces, distally on the legs and proximal on the tales of males, indicating that the initial lesion may have been associated with a behaviourally induced trauma. Recaptured animals (*n* = 43) during this period, provided records of lesion progression over time and while some healed up between capture events, others persisted for up to 24 months. Some turtles were repeatedly captured without lesions. Intra-species aggression associated with seasonal behaviours could potentially be the primary cause of skin trauma, followed by a secondary invasion of an unusual pathogen present in the environment.

## Introduction

Freshwater turtles are vulnerable to many human and natural factors and a systematic analysis of tortoise and freshwater turtle global distribution identified coastal Australia as one of three areas of priority for freshwater turtle conservation (1). In Australia, there are 25 species of freshwater turtles, 11 of which have a conservation status of vulnerable or worse (2). Most of the Australian freshwater turtles belong to the family Chelidae that retract their neck and head under the shell by folding it to one side and are therefore referred to as side-necks (3). They are a totem animal in some Indigenous Australian cultures and although they are collected and consumed, such traditional harvest is not of conservation concern (4). The major risks to Australia turtles include: invasive species, drought, habitat modification, and disease (2).

Due to their longevity and close association with the aquatic environment, freshwater turtles can be considered indicators of aquatic environmental health. In addition to being sentinel species for long term pollution exposure, they are also at risk of habitat loss or degradation, invasive species and diseases among other threats (5–8). The limited range of many Australian freshwater turtle species and the risk of extirpation from any of the abovementioned threats, means that conservation is a very real and urgent issue. Recently, the vulnerability of the range restricted Bellinger River Snapping Turtle (*Myuchelys georgesi*), became apparent when a novel disease affecting the turtles drove the population close to extinction in <1 month (9, 10) and highlights the need for a focus on factors affecting the health of Australian freshwater turtles.

In August 2016, cutaneous lesions were noted on a proportion of freshwater turtles captured as part of regular monitoring of wild living turtles in Alligator Creek, North Queensland. Here we report on the characteristics of the lesions, the spatial and temporal extent of the epidemic and the impact on the population through a longitudinal study.

## Materials and Methods

All handling of turtles were carried out under permits from James Cook University (JCU) Animal Ethics Committee (A2309) and Department of Environment and Science (WISP13270413 and WA0012830).

### Study Site

For the past six years in July/August the JCU Turtle Health Research Team has monitored a pond on Alligator Creek just outside the Bowling Green Bay National Park for freshwater turtle presence, demographics and general health. Alligator Creek originates in the Mt Elliott complex, inside the national park, where there is no industry, agriculture or human dwellings. The pond of interest, the Craill pond, is a hollow in the bedrock, ~30 m at the widest part, 90 m at its longest and 5 m at its deepest. Alligator Creek runs into and out of this pond. The Craill pond is inhabited by two species of turtles, *Emydura macquarii krefftii* and *Myuchelys latisternum* and a number of freshwater fish species as well as freshwater crustaceans and crocodiles (*Crocodylus johnstoni*). No unusual natural or anthropogenic event preceded the first observations of lesions in this population of turtles.

### Turtles

Approximately 50 turtles are captured annually by a combination of baited cathedral traps (passive sampling) and hand-capture (active sampling) to minimize any bias that might arise due to using only one method. *Emydura macquarii krefftii* tend to frequent the downstream reaches of large river systems or creeks, where they forage in the water and are considered omnivorous (11). *Myuchelys latisternum* are chiefly carnivorous and inhabit mainly the headwaters of rivers and tributaries, but can also be found in lagoons and billabongs (11). Both species can grow to 30 cm curved carapace length (CCL) and have a life-span of 20–30 years (12). In early August 2016, during the annual fieldtrip, cutaneous lesions on the soft tissue were recorded in the neck and tail region and on legs and feet of turtles (*n* = 15/50).

### Fieldtrips

Field monitoring of the Craill pond (Site 5) was continued at intervals during the next 2 years. Any turtle above 15 cm CCL was tagged with a small titanium tag (National Wing Tags, Jiffy 893) in the webbing of one of the hind-feet and re-captures could therefore be identified and disease progression recorded if relevant. Morphometrics of captured turtles and records of cutaneous lesions were collected from the turtles captured. The size at which male tails start to elongate varies between species and individuals (12). Turtles less than the median CCL of the 10 smallest identifiable males of each species were classed as juveniles, unless their tail was obviously differentiated (13). Physical examinations were performed on all captured turtles, this process included: freshwater leech counts, eye/oral/nasal examination, soft-tissue exam for swelling or bruising, activity level (high, medium, low), damage to shell or missing claws, and adult female turtles were palpated for eggs to assess breeding status. Disease investigation as well as water quality monitoring were also performed to develop an understanding of the etiology of the lesions.

### Spatial Pattern

In order to determine the spatial extent of this disease, turtle populations in four ponds upstream from Site 5 were also investigated during September/October 2016, starting with the pond at the highest elevation above mean sea level that the terrain allowed access to (Site 1). The five sites were at least 1 km apart and between 10 and 25 m difference in elevation (https://www.freemaptools.com/elevation-finder.htm), with Site 1 being at the highest elevation and the downstream sites gradually lower. See Table 1 for GPS location and elevation of study sites. To avoid spreading an un-identified agent upstream, we disinfected all equipment with 5% bleach after each site and moved in a downstream direction between sites on any given day. Turtle populations downstream from Site 5 were not investigated as it is assumed that they will be exposed via the flow to a given pathogen in the water (14). Additionally, the creek below this pond is frequented by saltwater crocodiles, which makes trapping and underwater hand capture too risky.

**Table 1:** GPS locations and elevation in meters (m) above mean sea level for the five sites investigated for lesions on turtles following the outbreak at Site 5 in July 2016.

**Site**	**Latitude**	**Longitude**	**Elevation**	**Description**
1	−19.4456	146.9746	95	Pond
2	−19.4417	146.9623	82	Pond
3	−19.4408	146.9541	69	Stream
4	−19.4367	146.9477	44	Pond
5	−19.429	146.9437	34	Pond

### Environmental Measurements

Conductivity, pH, and macro invertebrate counts and identity were recorded in the Craill pond when the lesions were first noticed (Site 5), as well as upstream from the outbreak. Air temperature historical data for the nearest weather station at Cape Ferguson was obtained from the Australian Bureau of Meterology (www.bom.gov.au) and mapped against proportion of turtles with lesions on the various fieldtrips.

### Data Analysis

All analysis was performed using the Statsmodels module (version 0.10.0) and Pandas library (0.24.2) in Python 3.6 (15, 16). Chi Square tests were used to compare the proportion on turtles (post-outbreak) with lesions in each species, sex and age class. The association of CCL with probability of having lesions (odds-ratio) was calculated by logistic regression. Pearson correlation was used to determine the relationship between mean maximum air temperature and the portion of animals with lesions grouped by month of capture.

### Laboratory Analysis

#### Pathology

With permission from relevant authorities, one male *E. m. krefftii* (14.0 cm CCL; 235 g), one male *M. latisternum* (14.8 cm CCL; 280 g) and one juvenile *M. latisternum* (12.0 cm CCL; 145 g) with lesions, were euthanized with MS222 according to Conroy et al. (17). A full necropsy and pathology investigation was performed, with emphasis on the lesions, but samples from all internal organs were collected from all three turtles. Samples were preserved in 10% neutral buffered formalin, embedded in paraffin, sectioned at 5 μm and stained with Haematoxylin and Eosin as well as Gomori's Methenamine-Silver (GMS), Ziehl-Neilsen (ZN), and Periodic Acid-Schiff (PAS) stains for examination (18).

#### Bacterial Culture and Initial Identification

Swab samples were collected in the field from 20 lesions of affected turtles and chins of 20 turtles without lesions. These areas were rinsed well with sterile saline before being sampled using dry swabs. Swab samples were also taken directly from liver, spleen, heart and heart blood during necropsy. All samples were immediately cultured on a range of media including Sheep Blood Agar (non-selective, aerobic and anaerobic) and MacConkey (selective for Gram negative bacteria) using standard culture methods. Bacterial identifications were determined using Biolog system and API20NE according to the manufacturer's instructions. All incubations were performed at 28 and 37°C for 48 h.

#### Bacterial Identification by Sequencing

Bacterial isolates that could not be identified using traditional methods were extracted to isolate genomic DNA using High Pure PCR Template Preparation Kit Version 20 (Cat. 11796828001 Roche, NSW) and Lysozyme (Cat. 10837059001 Roche, NSW) as per manufacturer's instructions. The concentrations of the resulting purified DNA samples were quantified using a Qubit 2.0 fluorimeter (Invitrogen) prior to storage in a −20°C freezer. The bacterial 16S rRNA gene was amplified with a set of universal primers, 27F and 1391R (5′-AGAGTTTGATCMTGGCTCAG-3′ and 5′-GACGGGCGGTGTGTRCA-3′; 1350bp) under standard PCR conditions consisting of an initial denaturation of 1 min at 95°C; followed by 30 cycles of denaturation at 95°C for 30 s; annealing at 55°C for 15 s; extension at 72°C for 15 s; and final elongation step at 72°C for 5 min. The PCR products were run on a 1.5% agarose gel to confirm amplification. Following confirmation, the PCR products were sent to Macrogen Inc., Seoul, South Korea for Sanger sequencing. Later, the nucleotide sequences were processed and aligned in Geneious (Biomatters Ltd.) followed by identification using NCBI nucleotide BLAST (https://blast.ncbi.nlm.nih.gov/Blast.cgi).

#### Blood Biochemical Parameters

Blood was collected in the field (1 ml) from the femoral vein of 20 turtles with and 20 without lesions using a 1 ml syringe and 27 gauge needle into a lithium heparin paediatric tube. The samples were transported back to the JCU Pathology Laboratory on ice. The samples were separated as soon as possible and the plasma samples stored frozen (−20°C) until analysed within 24 h on an automated Clinical Biochemistry analyser (Beckman Coulter AU480). The blood biochemical parameters were assessed and analysed using a student *T*-Test to determine significant difference in biochemical parameters associated with presence and absence of skin lesions and severity of infection.

#### Viral Culture

Samples from select organs (lung, spleen, liver, kidney and heart) from the turtles that underwent necropsy were stored at −80°C for viral isolation. Samples were homogenised with 1 ml Dulbecco's modified Eagle's medium (DMEM) supplemented with 100X Antibiotic-Antimycotic and subjected to three freeze/thaw cycles at −20°C before clarification by centrifugation at 12,000 rpm for 5 min. The swabs from lesions were soaked in the same culture medium and treated similar to the necropsy samples. A total of 500 μl supernatant from each sample was added to 80% confluent monolayers of FHM (fathead minnow) cells in a 24-well tissue culture plate (SARSTEDT®). The plates were incubated at 25°C and checked daily for cytopathic effects. Two blind passages were performed for each sample at weekly intervals, by transferring 100 μl of cell culture supernatant from inoculated wells to corresponding wells with new, non-infected FHM cell monolayers on a separate plate.

#### Viral Molecular Investigation

DNA extraction was carried out on swabs from lesions by NucleoMag® VET DNA isolation Kit (Macherey-Nagel) according to manufacturer's protocol. PCR was performed on lesion swabs using primers for various viral infections. The swabs were screened for herpesvirus, adenovirus, papillomavirus and ranavirus genome by PCR following the respective methods described by Vandevanter et al. (19), Wellehan et al. (20), Manire et al. (21), and Ariel et al. (22). Any samples that reacted in the assays were sequenced using BigDye v.3.1 Sanger sequencing (Macrogen, Korea) following gel extraction. Non-specific reactors (determined by sequence) were disregarded.

## Results

### Spatial Extent of Outbreak

During September / October 2016, 23 *M. latisternum* and four *E. m. krefftii* were captured at the four upstream sites investigated for spatial extent of the outbreak (Table 2). None of the 27 turtles caught upstream from the Craill pond had cutaneous lesions. *M. latisternum* was the only species found at the three sites at highest altitude. At the site closest to the Craill pond, both species were present. A gravid female was identified at sites 1, 3, and 4, indicating that the turtles were actively involved in reproduction and nesting during this time.

**Table 2:** Number, sex and age-class of *M. latisternum* and *E. m. krefftii* captured at upstream sites (1-4), during September/October 2016 fieldtrips to Alligator Creek.

	***E.latisternum***			***E. m. krefftii***	
**Site**	**Male**	**Female**	**Juvenile**	**Male**	**Female**
1	3	4^*^	1		
2	4	6	1		
3		2^*^	1		
4			1	1	3^*^

* *Indicates that one of the females captured were gravid with hard-shelled eggs*.

### Water Quality

The pH levels were close to neutral for all sites (mean pH: 6.96, 7.22, 7.46, and 7.1 for sites 1-2, 3, 4, and 5 respectively). The water conductivity values at all sites were low (mean μS/cm: 64, 43, 74, and 63 for sites 1-2, 3, 4, and 5 respectively). The macro invertebrate populations were different at Site 5 (the Craill pond). Site 1, 2, 3, and 4 all had highly sensitive macro invertebrates which indicates undisturbed water conditions, while Site 5 only had medium or lower sensitive invertebrates typically associated with creeks found near urban settings.

### Season and Proportion of Turtles With Lesions

**Figure 1:**
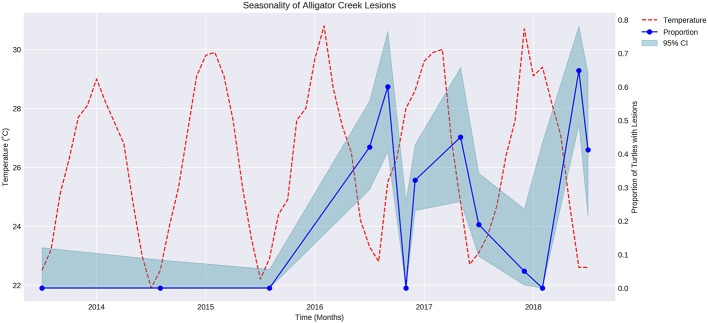
Fluctuation of mean maximum air temperature (red broken line) and proportion of turtles with cutaneous lesions at Alligator Creek (blue dots) at 95% CI (blue shading) over time from 2013 to 2018.

The proportion of turtles captured with lesions varied over time in a regular manner that appeared to be seasonal (Figure 1). Air temperature was overlaid on the graph showing proportion of turtles with lesions over time. The proportion of turtles with lesions is negatively correlated with the mean maximum air temperature during the sampling month (*P* < 0.05, *r* = −0.7).

### Gross Pathology

**Figure 2:**
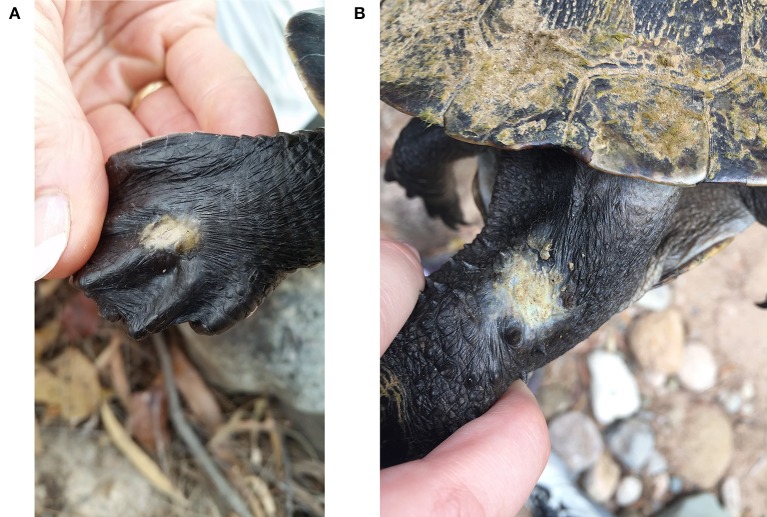
Typical appearance and location of skin lesions on turtles captured at Alligator Creek. **(A)** Skin lesions on top of foot of *E. m. krefftii*. **(B)** Skin lesion on neck of *M. latisternum*.

Lesions are characterized by focal areas of white-tan discoloration varying in size from 1 to 3 cm diameter, with irregular to rounded margins, which are occasionally firm, white and contracted, reflecting fibrosis (Figures 2A,B). The discolouration of the skin caused the lesions to look white against the darker skin and these white rounded patches were also noticeable when watching a turtle surface in the water. Position of 58 lesions on the skin of turtles were categorized using available photos from 22 females, 24 males, and 3 juveniles. Ninety three % of these type of lesions were on the dorsal surface of tails, legs and necks, the rest were lateral, except for lesions near the cloaca on two adult females. The lesions on the tails were mostly proximal (92%), predominantly distal on the legs (93%) and either proximal (43%) or middle of the neck (43%). Lesions were fairly evenly distributed between adult males and females for neck and leg lesions, but the tail lesions were recorded predominantly on males (92%). Turtles with lesions did not otherwise appear affected in terms of body condition and activity levels. The three turtles euthanized for necropsy were in good health with full gastro-intestinal tracts, plenty of body fat and no lesions other than the cutaneous lesions.

### Histopathology

**Figure 3:**
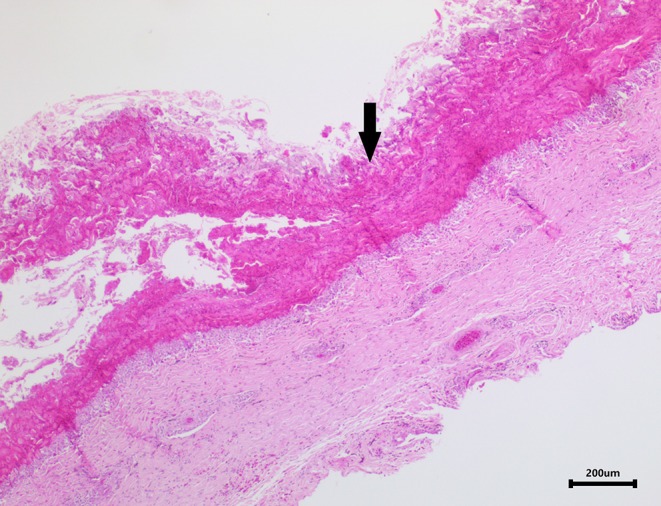
Image of turtle skin showing a dense serocellular crust (arrow) overlying the severely ulcerated epidermis.

**Figure 4:**
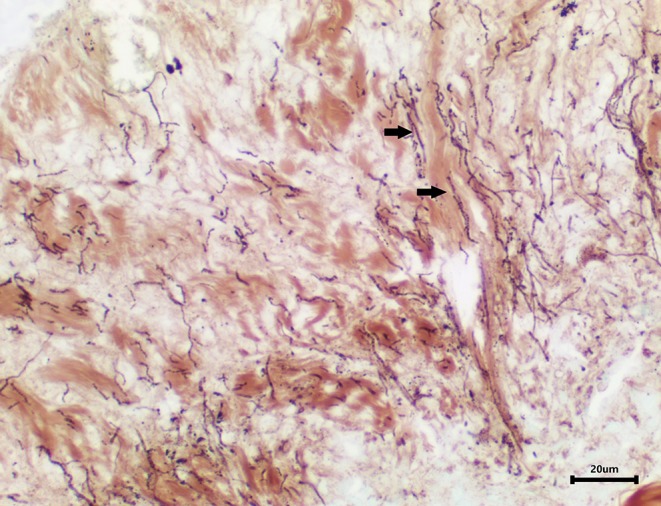
Area of epidermal necrosis and ulceration embedded with filamentous organisms (GMS stain).

Skin samples from all three turtles were similar, with each demonstrating a severe, subacute to chronic, multifocal, necrotizing, suppurative epidermitis and dermatitis. The epidermis was multifocally eroded to ulcerated and overlain by a dense serocellular crust embedded with colonies of coccobacilli and rods (predominantly Gram negative) (Figure 3) and filamentous organisms (Gomori's methenamine silver stain (GMS) positive) (Figure 4). No organisms were observed with Ziehl-Neelsen stain. In some areas there was also a layering effect of keratin and exudate. At the dermo-epidermal junction, there was a band of inflammation and often, granulation tissue; the former consisted largely of granulocytes and macrophages. In the dermis, there was patchy infiltration of granulocytes and small blood vessels were congested and frequently cuffed by moderate numbers of lymphocytes and plasma cells. Vascular fibrinoid degeneration was occasionally observed, but rare. In the adjacent intact epidermis, there was frequently spongiosis of the basal cell layer. No significant abnormalities were noted in other organs.

### Bacteriology

Bacterial culture of swabs from internal organs during necropsy were negative, while a number of bacterial strains were cultured and identified from swaps of normal skin in turtles with and without lesions as well as directly from lesions (Table 3).

**Table 3:** Number of bacterial strains isolated for 14 species of bacteria from 20 turtles without lesions and normal skin and lesions in 20 diseases freshwater turtles.

	**Turtle without lesion**	**Turtle with lesion**	
	**Normal skin**	**Normal skin**	**Lesion**
*Serratia marcescens*	2	3	9
*Staphylococcus capitis*	1	2	4
*Fictibacillus* and *Bacillus*	1	1	4
*Aeromonas sobria*		2	3
*Aeromonas hydrophila*		4	1
*Chryseobacterium*			1
*Acinetobacter*		3	
*Aquitalea*		1	
*Diaphorobacter* and *Acidovorax*		1	
*Microbacterium* sp.		1	
*Pseudomonas fluorescens*		1	
*Bacillus*	1		
*Citrobacter youngae*	1		
*Pseudomonas aeruginosa*	1		

### Blood Biochemical Parameters

No statistically significant differences were observed in the blood parameters of turtles from different species, age groups, sex, sites, and presence or absence of lesions.

### Virology

There was no development of CPE in cell cultures after three blind passages and the PCRs to detect ranavirus, herpesvirus, adenovirus, and papillomavirus genome were also negative for the samples collected.

### Turtles Captured

**Figure 5:**
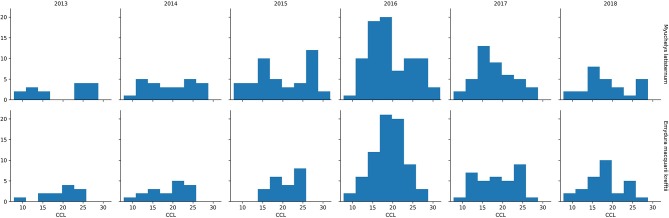
Number of *M. latisternum* and *E. m. krefftii* recorded in each size class of curved carapace length (CCL) from 2013 to 2018.

In the period from July 2013 to July 2018, 249 *M. latisternum* and 192 *E. m. krefftii* were captured in Alligator Creek at Site 5 (Table 4). All size groups of turtles were continuously captured over the period for both species (Figure 5).

**Table 4:** The total number of *M. latisternum* and *E. m. krefftii* captured as well as those presenting with lesions in the Craill pond, during fieldtrips to Alligator Creek at various dates from 2013 to 2018.

**Date**	***M. latisternum***		***E. m. krefftii***	
	**Total**	**Lesions**	**Total**	**Lesions**
21/07/2013	15	0	13	0
10/08/2014	25	0	17	0
2/08/2015	44	0	21	0
31/07/2016	26	12	24	9
10/09/2016	13	10	12	5
6/11/2016	10	0	1	0
10/12/2016	45	17	39	10
5/05/2017	13	5	7	4
30/07/2017	21	4	16	3
10/12/2017	9	1	11	0
10/02/2018	1	0	4	0
28/06/2018	18	11	19	13
29/07/2018	9	2	8	5
	249	62	192	49

The Curved Carapace Length (CCL) of turtles captured ranged in size from 7.8 to 30.0 cm for *M. latisternum* and 8.4 to 28.5 cm for *E. m. krefftii*. For both species, juveniles comprised approximately 1/3 of the captured turtles, while females dominated the *M. latisternum* captures (ratio male: female = 54 : 119) and males the *E. m. krefftii* turtles (male: female = 99 : 39) (Table 5).

**Table 5:** Male, female and juvenile *M. latisternum* and *E. m. krefftii* captured at Alligator creek from 2013 to 2018.

	***M. latisternum***	***E. m. krefftii***
Males	54	99
Females	119	39
Juveniles	76	54

### Presence of Lesions Over Time

**Figure 6:**
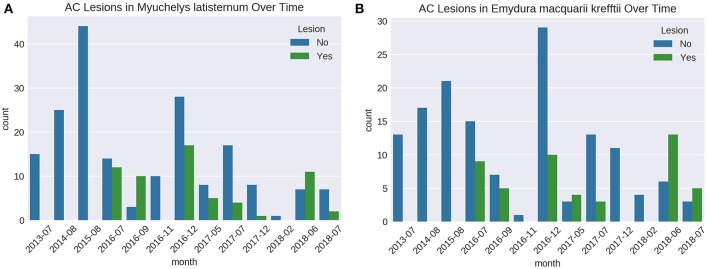
Number of captured *M. latisternum* **(A)** and *E. m. krefftii* **(B)** turtles without cutaneous lesions (blue) and with lesions (green) on fieldtrips to Alligator Creek from 2013 to 2018.

Cutaneous lesions were not recorded in this sub-population of freshwater turtles before July 2016, nor in any other freshwater turtles studied in the region, before or since. The number of turtles caught on each fieldtrip varied, as did the proportion of animals with lesions (Figures 6A,B). Presence of cutaneous lesions on turtles captured on fieldtrips since July 2016 varied from 0 to 77% for *M. latisternum* and 0 to 68% for *E. m. krefftii*.

Considering data from the first records of the outbreak in July 2016 and onwards from Site 5 only, there was no significant difference in proportion of turtles with cutaneous lesions between the two species (*X*^2^ (1, *N* = 306) 0.262296; *p* > 0.05), nor was there any significant difference between sex in adult *M. latisternum* (*X*^2^ (1, *N* = 108) 0.908654; *p* > 0.05), nor for *E. m. krefftii* (*X*^2^ (1, *N* = 102) 0.728571; *p* > 0.05).

There was however, a significant difference in proportion of turtles with lesions between adults and juveniles (*X*^2^ (1, *N* =3 06) 16.67154; *p* < 0.001), with juveniles being less likely to have lesions than adults for the two species combined. For a 1 cm increase in CCL there is a 15.2% (*p* < 0.001, 95% CI 8.9–21.9) increase in odds of having a lesion.

### Lesion Progression Over Time

**Figure 7:**
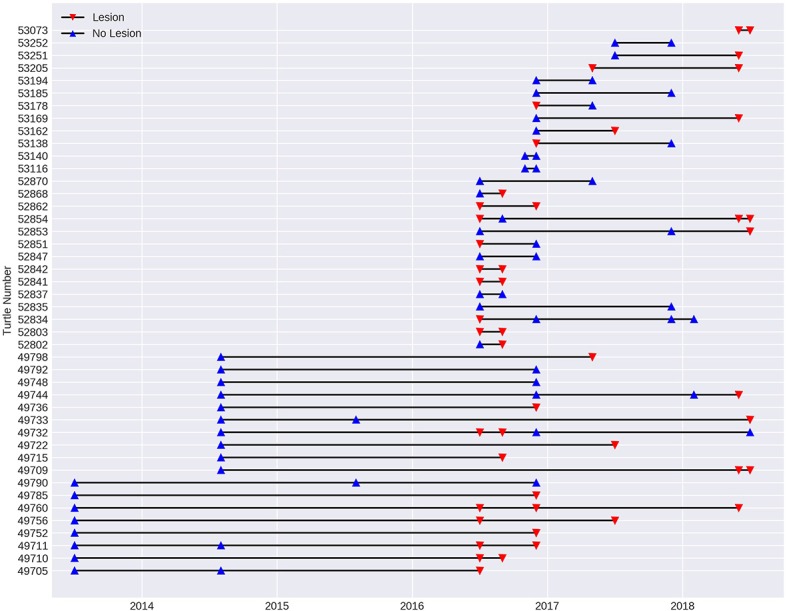
Presence of lesions (red triangle) and no lesions (blue triangle) in the 43 turtles recaptured at Site 5, Alligator Creek between 2013 and 2018. Individual turtles are identified with tag number.

In order to assess the progression of the disease over time, a sub-set of the data from 2013 to 2018 was investigated using turtles (*n* = 43) that had been recaptured since the initial recorded outbreak in July 2016. Spanning 11 fieldtrips over 2.5 years, 15 turtles remained without lesions on subsequent fieldtrips spaced from 2 to 30 months apart. Two of these turtles were previously recorded with lesions, but had recovered. Fourteen turtles that were registered with lesions, still had lesions 2–24 months later. One of these turtles (number 52854, Figure 7) was initially registered with lesions, which appeared to heal over a 3-month period, but it was recaptured with lesions 22 and 24 months later. Eight of the recaptured turtles were initially registered without lesions, but were recapture with them between 3 and 24 months later. On the other hand, five turtles with lesions were found to have healed on subsequent fieldtrips 2 to 5 months later (see Figure 7).

## Discussion

Several threats to freshwater turtles have been identified as contributors to their decline, including urban development (23–26) and introduced predators (4, 27), however, the study on the impact of disease on wild animals including populations of freshwater turtles is hampered by their cryptic and often remote habitats (22). Occasionally, spectacular outbreaks in public areas draw the attention of the general public and scientists alike and spur in-depth investigations with a multi-disciplinary approach (9, 10). The appearance of cutaneous lesions on a proportion of freshwater turtles captured as part of regular monitoring of wild living turtles in Alligator Creek, North Queensland was likewise an opportunity to study the course of disease development in a wild population over time.

### Spatial and Temporal Extent of the Epidemic

The epidemic was confined to a natural pond on Alligator Creek, the Craill pond (Site 5), and although turtles were found upstream from the pond, these did not have lesions during the initial stages of the outbreak in 2016. Downstream populations were not investigated due to presence of saltwater crocodiles and associated risk, but were considered infected according to general principles guiding zoning for diseases in streams (14). Prior to July 2016, there were no records of such lesions in this area or other streams or rivers in the region despite regular annual sampling, but turtles were continuously caught with lesions in this pond for the 3-year monitoring that followed.

Water quality parameters tested did not indicate an acute contamination event although the Craill pond was considered more disturbed than the upstream sites, based on the macro invertebrates found there. However, it was not possible to completely rule out a pollution, contamination, or other toxic event as water was only sampled at one timepoint (during the initial outbreak).

An inverse relationship between temperature and the proportion of turtles captured with lesions throughout the year was identified. The relationship may not be a direct one, but temperature fluctuate with season and other seasonal factors such as migration and aggression in response to reproduction, basking sites or food availability (28) may also have increased the risk in this pond at certain times of the year.

### Characteristics of the Lesions

Histological examination revealed that the condition was largely confined to the epidermis, while all other organs in the animals investigated appeared normal. Skin lesions associated with ranaviral infection have been reported in lizards and turtles, but in most cases internal organs were also infected (29–32). While there was no detectable bias toward sex or species of turtles, the odds ratio indicates that the larger the turtle, the higher the risk of a lesion, and juveniles were rarely affected. The position of the lesions on the turtles indicated a non-random distribution on the skin and it is therefore likely that the initial skin lesion may have been caused by trauma and associated with a behavior where certain parts of the body would be at higher risk. The lesions were predominantly dorsal indicating that the trauma may have originated from above the turtle rather than below. The lesions on legs were mostly distal, while neck lesions were both proximal and middle of the neck. Tail lesions were essentially all proximal and on males apart from lesions near the cloaca on two females.

Given that peak lesion prevalence (May-September) precedes the nesting season of in this region, which is October to January for *E. m. krefftii* and September to March for *M. latisternum* according to Cann (12) and as evidenced by hard-shelled eggs detected via palpation on fieldtrips, it is possible that courtship behavior would put adult turtles in a higher risk group, either through intra-species aggression during courtship and mating (28) or because this behavior favors another risk factor such as a predator. Biting during courtship and mating could also explain the lesions on the male tails as they may be targeted by competing males as seen in other freshwater turtles and sea turtles (28, 33). Alternatively, the males have larger tails than the females and would therefore be an easier target, but lesions on legs were mostly distal and on the feet, while the tail lesions were primarily proximal, which indicates that male tails are somehow a different target from legs. The lesions on the cloaca of two females could be associated with skin trauma during mating.

Courtship and mating is a natural occurrence and by itself would not cause such lesions. The investigation therefore focused on identifying a unique infectious agent, which could have gained entry following skin trauma.

### Agent Causing the Lesions

Occasionally there are reports of wild populations of turtles succumbing to unknown pressures: several hundred *E. macquarii*, for instance, were unaccountably found dead at Lake Boga, South Australia, during the first half of 1976, while many others were clearly emaciated (12). Such mass mortalities are possibly caused by intoxication or infectious diseases. Most publications concerning the influence of disease on freshwater turtle populations in Australia are based on mass mortality events or general health problems in a population (7, 34). It is therefore challenging to interpret the presence of microorganisms in sick turtles as pathogenic or commensal due to lack of baseline data.

A number of bacterial strains were cultured and identified from swaps of normal skin in turtles with and without lesions as well as directly from lesions. The only bacteria isolated only from a lesion and not normal skin on any of the turtles was *Chryseobacterium*. This was only isolated in one instance, and is therefore not considered a causative agent. Some of the other bacteria are normal skin flora (*Aeromonas sobria, Aeromonas hydrophila, Serratia marcescens, Staphylococcus capitis, Fictibacillus*, and *Bacillus*), but they can on occasion turn pathogenic (22). In this study they were isolated at a higher rate from lesions and could therefore be secondary pathogens. There seemed to be a different composition of bacteria on skin of some turtles with and without lesions, but again, they do not appear to have any causation effect.

The blood biochemical parameters in turtles with lesions did not differ from turtles without lesions and none of the swab samples reacted in the virological assays for ranavirus, herpesvirus, adenovirus, or papillomavirus, which have been reported in reptiles on previous occasions (35). Ranaviral infections in turtles have been associated with intra-cytoplasmic inclusion bodies (36, 37), but these were not detected. A toxic etiology would also seem unlikely in this case, based on the distribution of the lesions, as it would be expected that the lesions would be random and affect all areas of the animal, rather than just the dorsum.

### The Impact on the Population Through a Longitudinal Study

The lesions in the turtles at Alligator Creek, did not appear to have a dramatic impact on their overall health over the 3-year monitoring period following the first appearance of the lesions. There was no obvious decline or change in the demographics of the turtle populations and some turtles recorded with lesions were recaptured the following years, indicating that the affliction was not definitively lethal, but slow progressing. In addition, the post mortem examination following euthanasia of three adult males in clinically good health, but with skin lesions, revealed a good amount of body fat and a gastro intestinal tract full of ingesta, indicating that the animals were thriving, despite the winter temperatures and presumed mating season.

Field monitoring was intensified in the months after the initial reports of lesions so there are more data from late 2016 than other years, and fieldtrips in 2016–2018 were also spaced over more months and seasons. Following the initial outbreak in 2016, we continued to capture turtles of all sizes and both species of turtles, including recapture of 43 tagged turtles. This sub-sample of recaptured turtles was very useful in defining disease progression in the population. It revealed that some turtles stayed free of lesions over several years even if they co-existed with animals with lesions. Some turtles were repeatedly registered with lesions over several fieldtrips, while others had healed between captures. Other turtles were initially registered without lesions, but developed them between subsequent fieldtrips. Future monitoring will be required to determine if the population will fully heal over time.

This pattern of disease progression suggests that the agent is not a lethal one and that healing may occur naturally. It also suggests that exposure to the agent in the environment is not sufficient to cause disease, but there must be a contributing risk factor involved to create a portal of entry in a non-random fashion. These findings fits into the theory of an initial skin trauma associated with certain seasons, followed by entry and establishment of a pathogen present in the local environment. The seasonal aggression associated with courtship and mating could potentially be the cause of the skin trauma, but the identity and drivers for the appearance of a unique pathogen in this population is still under investigation.

## Data Availability Statement

The datasets generated for this study are available on request to the corresponding author.

## Ethics Statement

The animal study was reviewed and approved by James Cook University Animal Ethics Committee.

## Author Contributions

EA and WW contributed conception and design of the study. All authors contributed to the acquisition, analysis and interpretation of data for the work. WW and KD organized the database and photos. WW performed the statistical analysis. EA wrote the first draft of the manuscript. All authors wrote individual sections of the manuscript and revising it critically for important intellectual content and approved the submitted version.

### Conflict of Interest

EE was employed by the company Ausphage. The remaining authors declare that the research was conducted in the absence of any commercial or financial relationships that could be construed as a potential conflict of interest.
